# Computational inference and analysis of genetic regulatory networks via a supervised combinatorial-optimization pattern

**DOI:** 10.1186/1752-0509-4-S2-S3

**Published:** 2010-09-13

**Authors:** Binhua Tang, Xuechen Wu, Ge Tan, Su-Shing Chen, Qing Jing, Bairong Shen

**Affiliations:** 1 Department of Bioinformatics, Tongji University, Shanghai, 212003, China; 2 Institute of Protein Research, Tongji University, Shanghai, 212003, China; 3 Department of Computer Science, ETH, Zurich, 8092, Switzerland; 4 CISE and Systems Biology Lab, University of Florida, Gainesville, FLA, 32611, USA; 5 Institute of Health Sciences, Shanghai Institutes for Biological Sciences, Chinese Academy of Sciences, Shanghai, 200031, China; 6 Center for Systems Biology, Soochow University, Suzhou, 215006, China

## Abstract

**Background:**

Post-genome era brings about diverse categories of omics data. Inference and analysis of genetic regulatory networks act prominently in extracting inherent mechanisms, discovering and interpreting the related biological nature and living principles beneath mazy phenomena, and eventually promoting the well-beings of humankind.

**Results:**

A supervised combinatorial-optimization pattern based on information and signal-processing theories is introduced into the inference and analysis of genetic regulatory networks. An associativity measure is proposed to define the regulatory strength/connectivity, and a phase-shift metric determines regulatory directions among components of the reconstructed networks. Thus, it solves the undirected regulatory problems arising from most of current linear/nonlinear relevance methods. In case of computational and topological redundancy, we constrain the classified group size of pair candidates within a multiobjective combinatorial optimization (MOCO) pattern.

**Conclusions:**

We testify the proposed approach on two real-world microarray datasets of different statistical characteristics. Thus, we reveal the inherent design mechanisms for genetic networks by quantitative means, facilitating further theoretic analysis and experimental design with diverse research purposes. Qualitative comparisons with other methods and certain related focuses needing further work are illustrated within the discussion section.

## Background

Various cell phenotypes and functions within multi-cellular organisms relate directly to genetic contents decoded from DNA and RNA during transcriptional and translational processes. Inference of gene regulatory networks or maps for those intercellular processes plays significant roles in the further comprehension of underlying regulatory mechanisms. Thus reconstructing such biological regulatory networks directly from gene profile datasets measured at different cell phases, types and even species becomes one of the foremost research topics recently.

Due to capabilities of simultaneous measurement for multiple expression profiles with gradually increasing accuracy and decreasing costs of experiments, those advances in high-throughput microarray and ChIP assays techniques facilitate the corresponding learning and inference of the regulatory maps and even functionality of these genetic networks. During the past decades, manifold inference and learning methods have been proposed to integrate raw data to computational frameworks for network models, such as (probabilistic) Boolean network and (dynamic) Bayesian network, systematic differential/difference equations [1-6], information theory-based modelling [7-10], graph and control theoretic approaches [11-13].

Furthermore, most of current biochemical networks are regarded as static descriptions of the inherent regulatory mechanisms in the sense that once the system models and parameters for those genetic networks are set, the regulatory processes are determined. While during genetic transcriptional and translational processes, real-world regulatory maps may undergo various perturbations from intercellular and intracellular signals and undiscovered factors. From this perspective, a single modelling mode may not be sufficient to characterize all kinds of possible structures of these networks, or even crucial ones for specific analysis purposes. The problems above solicit flexible mechanism designs to improve the present rigid methods for network inference.

Within the following parts, we propose an integrative supervised learning method for the inference of time-delayed cell cycle regulatory mechanism based on information and signal processing theories. We firstly introduce definitions for those crucial concepts as correlation measure and mutual information; then we propose a novel associative quantity for the two kinds of dependency measures. With the proposed integrative metric and the *P*-values from the Pearson correlation operations on all pairwise genes from the raw data pool, we may determine the dependency and connectivity among those pairwise candidates. Such kind of integrative dependency metric improves the performance of above one-fold linear or nonlinear criterion since multiple-criteria may perform cross validation functions for measuring dependency within the test results.

Moreover, from signal processing theory [5,14-16], a phase-shift metric is introduced for measuring time delay of gene expression within pairwise candidates. The advantages of such a phase-shift metric lie in its flexible characteristics of determining the regulatory delay variation via dynamic thresholds of relevant transfer gains between pairwise candidates. Since factual regulatory mechanisms possess multiple possibilities during biological processes and underlying regulatory delay effects may vary in the context of related courses. The phase-shift metric elucidates such possibilities underlying the regulatory mechanisms quantitatively via dynamic threshold of transfer gains.

The other advantage of the method includes its inherent capabilities of integrating existing biological knowledge as *a priori*. This kind of knowledge-based inference method avoids redundant false-positive connectivity within pairwise gene candidates. Moreover, dynamic threshold for transfer gain facilitates its potential applicability to the majority of problems facing theoretic and experimental biologists. Since regulatory connectivity underlying pairwise gene candidates may differ from each other at various tissues and sampling times, quantitative determination of these regulations with existing empirical and theoretical knowledge will act as much more effective roles, compared to most of current simplex computational approaches.

## Results

The supervised learning framework mainly covers two aspects, namely, it should characterize pairwise regulatory strengths and constrain subsequent computational redundancy. We utilize the proposed method for two real-world datasets, selected from the Stanford Microarray Database. The both datasets are of different statistical characteristics, normalized and benchmarked in the recent literatures [17-19].

### Analysis on the *Saccharomyces cerevisiae* cell cycle microarray dataset

**Figure 1:**
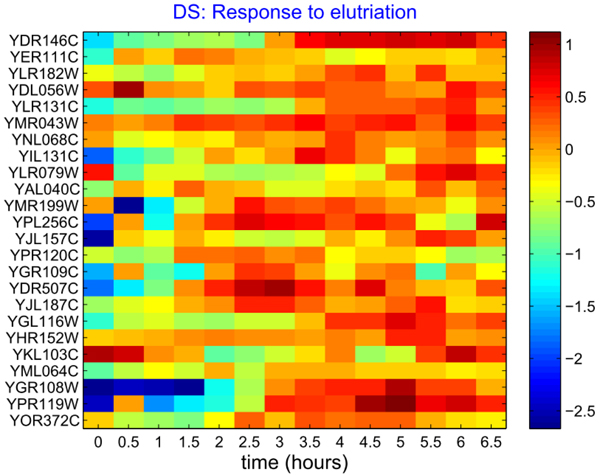
**The log2-normalized gene expression profile for 24 genes from the cell cycle regulatory network (Experiment condition: response to elutriation).** The horizontal coordinate represents the sample time. (14 points from 0 to 6.5 hours, equally sampled per 30 minutes); the vertical coordinate illustrates 24 genes from the cell cycle genetic network.

The first *Saccharomyces cerevisiae* cell cycle microarray dataset was measured through the regulatory responses under the elutriation treatment, available at the Stanford Microarray Database. The dataset has been benchmarked in the literature [10,20,21]. The log2-normalized expression profile of 24 genes from the regulatory network is plotted in the following Figure 1.

Based on the definitions and concepts illustrated in the methodology part, we calculated the mutual information, correlation and *P*-values among pairwise genes for constructing regulatory activities. The mutual information matrix, correlation and corresponding *P*-values are given in the additional Figure 1-A in Additional file 1 and additional Figure 1-B in Additional file 2.

As depicted in the lower sub-graph of the additional Figure 1-B in Additional file 2, there are more than 101 pairs with their *P*-values not greater than 0.05 (indicated by the vertical line), commonly adopted in most research fields. Therefore around 60% or 165 hypothetic reaction edges are redundant and may be reduced for the further reconstruction of the regulatory network, and thus in this map, on average, every gene has direct or indirect relations with 4 to 5 other genes. The phenomena conform to the generally recognized viewpoints that most biochemical regulatory networks are sparsely constructed.

**Figure 2:**
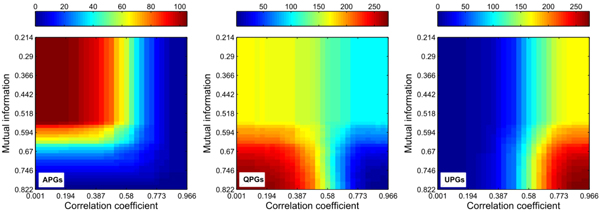
**The global statistics for pairwise gene numbers under different mutual information values and correlation coefficients.** Totally, there are 276 pair candidates for the network of 24 genes. The horizontal axis represents different mutual information thresholds, and the vertical axis illustrates correlation coefficient thresholds. The corresponding three-dimensional graph is given in the additional Figure 2-A in Additional file 3 for comparative purposes within the three groups.

Thus through dynamic thresholding of mutual information and correlation coefficient, we obtain the global distributions for three pair groups under dynamic metrics. The distributions for the classified pair groups are illustrated in Figure 2. 

The supervised inference procedure starts from the respective centroids, *i.e.* 0.5709 and 0.4358 for mutual information and correlation coefficient. Actually, from the heat maps illustrated in Figure 2, we may find the proximately-diagonal symmetries of the variations between mutual information and correlation coefficient, especially for the group APGs. Such interesting phenomena facilitate detecting suitable initial thresholds and optimal iteration tracks.

Also with the acquired knowledge, *e.g.* the genetic networks are sparsely constructed and their topologies normally follow the ‘small-world’ properties, the interactive computations halt at 0.4950 for mutual information and 0.4602 for correlation thresholds. At the terminated thresholds, the APGs, QPGs and UPGs groups have 83, 157 and 36 candidates respectively.

**Figure 3:**
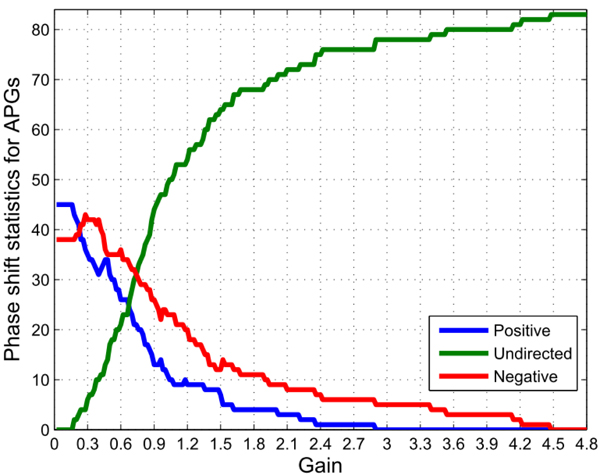
**The global phase-shift statistics distribution for the APGs of the cell cycle regulatory network (totally 83 pairwise candidates in APGs).** The phase-shift statistics vary as functions of the gain thresholds. The blue bold curve represents the integral tendency of gene pairs with leading phase shifts (positive), the red for the pairs with lagging phase shifts (negative), and the green for those without detected phase shift (undirected), *i.e*. there might be no regulatory activities between corresponding gene pairs (the same as in following figures). Through dynamic gain thresholding, we may easily determine concrete regulatory time lags, regulatory directions and signal intensities from the quantitative signal processing perspective.

Thus, we might calculate the global phase-shift statistics for the APGs group, based on the signal processing theory defined in the methodology section. Figure 3 illustrates the calculated global phase-shift statistics. The details of the statistics for the gene pairs in the APGs group are given in the additional Figure 3-A in Additional file 4.

**Figure 4:**
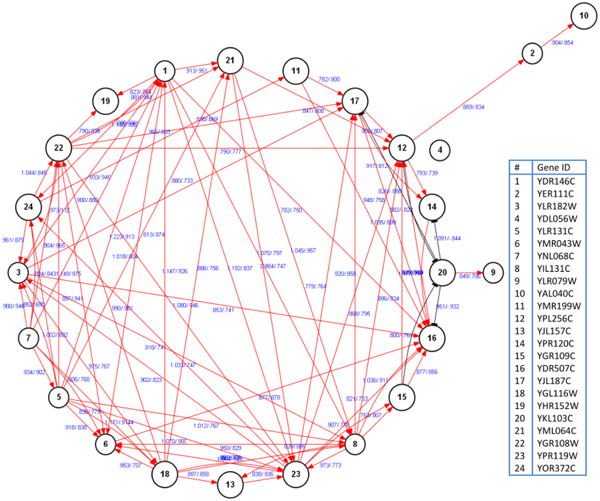
**The interweaved cell cycle regulatory network rebuilt based on the MICORPS framework.** Each gene/protein is denoted as a black-edged circle. The calculated associativity metric and phase-shift information between pairwise genes are marked as blue along each bilateral links, see the additional Figure 4-A in Additional file 5 of associativity measure for details.

For this case, the gain threshold is set at 0.3, see the additional Figure 3-A in Additional file 4.  The centroids for the mutual information and correlation coefficients within total available pairs are 0.6193 and 0.6900 respectively. The whole searching for optimal solutions stops with the mutual information (0.4950), correlation coefficient (0.4602) and *P*-value (0.05). Thus we get valid links and concrete regulatory directions at the current conditions. Figure 4 illustrates the reconstructed regulatory network.

As depicted, only the gene #4 (YDL056W) is isolated from the network structure, meaning that YDL056W might belong to other regulatory processes at the current situation. Besides, the gene #2 (YER111C) only has a single regulatory link, similar to the genes #9 (YLR079W) and #10 (YAL040C). While for such genes as #1 (YDR146C), #3 (YLR182W), #16 (YDR507C), *etc*., they have multiple regulatory links, indicating they undertake much more responsibilities during the underlying interaction and regulation processes.

Since the above analysis is for the case of normal statistical characteristics, one may directly utilize the proposed methods. Within the following part, we discuss another kind of microarray dataset of different statistical properties.

### Analysis on the dataset from a p53 pathway with multiple feedback loops

**Figure 5:**
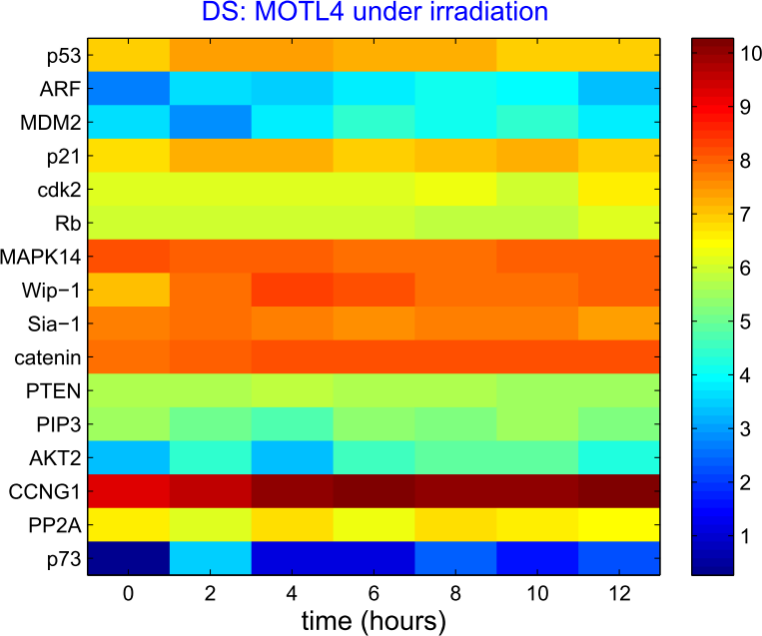
**The triplicate MOTL4 microarray experiments are implemented under irradiation from 0 to 12 hours at intervals of 2 hours.** The expression profile is plotted with the mean values of the triplicate datasets. The horizontal axis denotes the time range from 0 to 12 hours, and the vertical axis for the corresponding 16 gene/protein names.

The profile dataset of the p53 pathway with multiple feedback loops is selected from the recent work [10], concerning human leukaemia cell lines (MOTL4) with the functional protein p53. The triplicate MOTL4 microarray experiments are implemented under irradiation from 0 to 12 hours at intervals of 2 hours, depicted in Figure 5. The additional Figure 5-A in Additional file 6 and additional Figure 5-B in Additional file 7 illustrate related mutual information matrix and correlation statistics of total gene pairs for the p53 pathway.

However, this kind of dataset does not satisfy the above network-constructing algorithm since there are only 10 pair candidates with their *P*-values below 0.05 (91.7% of the total pairs with correlation statistical significance above 0.05), see the additional Figure 5-B in Additional file 6. Therefore, it is impossible to construct a genetic network of 16 genes with just 10 suitable candidate links under the current situation. Thus, before utilizing the PGHC algorithm, it is necessary to modify the *P*-value threshold.

**Figure 6:**
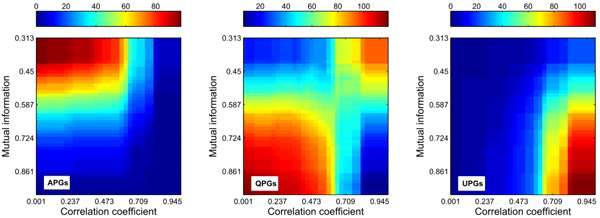
**The global statistics for pairwise gene numbers under different mutual information values and correlation coefficients.** Totally, there are 120 pair candidates for the network of 16 genes. The horizontal axis represents different mutual information thresholds, and the vertical axis illustrates correlation coefficient thresholds. See the additional Figure 6-A in Additional file 8 of three-dimensional comparative graph for the three groups.

As the former case, 40%~45% of the total pairs as suitable candidates are needed for constructing genetic networks, then we lift the threshold higher enough, and derive necessary suitable pair candidates for composing the group APGs via the proposed PGHC algorithm. For this case, we lift the *P*-value threshold to 0.8 or so, and obtain the global statistical distribution for three groups through dynamic threshold of mutual information and correlation coefficient. The distribution plots for the classified pair groups are illustrated in Figure 6.

**Figure 7:**
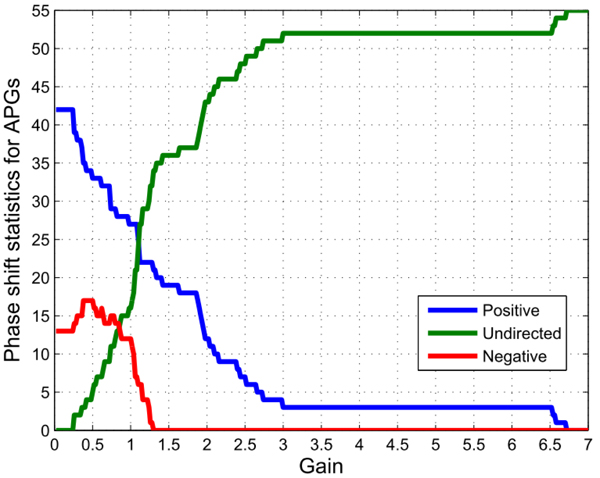
**The calculated phase-shift statistics distribution (totally 55 pairwise candidates for the APGs group in the multi-feedback p53 pathway).** The blue bold curve represents the integral tendency of gene pairs with leading phase shifts (positive), the red for the pairs with lagging phase shifts (negative), and the green for those without detected phase shift (undirected), *i.e*. there might be no regulatory activities between corresponding gene pairs.

Thus, we might calculate the global phase-shift statistics for the APGs group, based on the signal processing concepts defined in the methodology section. The calculated global phase-shift details are given in Figure 7. The additional Figure 7-A illustrates the details of the statistics for the gene pairs in the APGs group.

Within the following network-building procedure, we still choose the corresponding centroids of both metrics as the initial points for the iterative computation. The centroids for the mutual information and correlation coefficients for the totally available pairs are 0.7992 and 0.5203 respectively.

**Figure 8:**
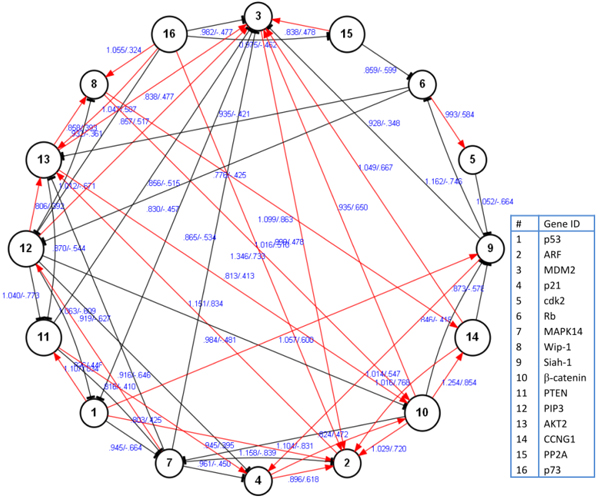
**The constructed genetic graphs under different gain thresholds.** The structure is constructed with gain threshold at 0.3, and the additional Figure 8-A in Additional file 10 adopts 1 as the gain threshold. As depicted in the figure, #5 (cdk2) is the weak-connected node, #3 (MDM2), #10 (β-catenin), and #12 (PIP3), *etc*. are the strong-connected ones under the current gain threshold.

The searching for optimal solutions stops when the mutual information threshold backtracks to 0.7 and the correlation coefficient takes 0.3 and the *P*-value adopts 0.8 for the whole iterative procedure. To testify the significance of gain to network topological structures, the gain thresholds take 0.3 and 1 respectively. Thus, we may derive valid links and concrete regulatory directions at the two gain thresholds from the additional Figure 7-A in Additional file 9. And the reconstructed regulatory networks are plotted in Figure 8 and the additional Figure 8-A in Additional file 10. The detailed information for the related links within the APGs group is given in additional Figure 8-B in Additional file 11.

## Discussion

### The comparison with the currently-available inference methods

Currently, there exist several inference approaches for the biochemical networks, *e.g.* probabilistic approaches, equation-based methods, *etc*. As depicted within the context, the proposed method tackles such key inference issues for integrating previously-acquired biological knowledge as *a priori* via dynamic threshold of multi-source information. Thus compared with most computation-oriented methods, the proposed inference framework ameliorates inference accuracy and experimental achievements within a problem-oriented scheme.

Secondly, the proposed method tackles one of most important problems from the perspective of signal processing theory, namely, the determination of regulatory directions between candidate gene pairs. The introduced metrics quantify those underlying regulatory strengths, directions between pair candidates globally and comparatively. Thus, it facilitates the follow-up network-rebuilding procedure.

Moreover, the proposed inference framework might illustrate in parallel multiple optimal or suboptimal potential regulatory maps, instead of the one computational solution for one problem scheme, since for most cases such solutions cannot explain convincingly so much inherent mechanism as expected. The proposed method might utilize the diverse knowledge available, either from concrete biochemical experiments or current literatures.

### The current focuses of the proposed method and its future directions

Although the proposed inference framework is validated with the real-world profile datasets, there are still several directions needing further refinement, depicted within the below section.

In practice, most available profile datasets are of high dimensions, particularly as those kinds of less-point and multi-sample profiles, together with unavoidable measurement noises, *etc*. Thus, any suitable pre-processing is demanded for the kinds of subjects before further analysis. The indispensable pre-processing covers de-noise treatments, functional and hierarchical clustering and so forth, before the next-stage network reconstruction.

The second concern mainly relates to the biologically-functional analysis on relative network modules and motifs by quantitative means. The proposed framework deciphers genetic regulatory activities with a rich-information mode. Thus, the inference results and related information between pairwise candidates have the potentials for those applications as succeeding identification of biological modules and motifs of particular interests.

The third focus might go to topological properties of inferred regulatory networks. Quantitative analysis and comparison between diverse constructed topologies might reveal inherent coordination and organization mechanisms, which thus have potential applications in, to name a few, identifying target genes, and novel drug discovery, particularly for those subjects in computational systems biology.

## Conclusions

Within the work, we propose a combinatorial theory-based learning pattern for the inference and analysis of genetic networks from microarray time-series datasets.

For different kinds of microarray datasets gathered from multiple organisms and species, there still does not exist such an efficient solution applicable to most of current problems facing biological theoreticians and experimentalists. In consideration of previously-acquired knowledge, decision-makers’ preferences and practical constraints, the network inference might be transformed into a kind of multi-objective combinatorial optimization (MOCO) problem.

Compared with currently available methods for inferring biochemical networks [20,21], the proposed approach renders the possibilities for biologists to incorporate concrete theoretic and empirical knowledge, and thus to construct regulatory networks with much more reliabilities and accuracy. Secondly, different regulatory models should focus on specific perspectives and utilities adopted by the builders, thus the inherent complexity from the inference procedures and the necessity to optimize those results appeal such a kind of associative relevance metrics and multiobjective combinatorial optimization method.

To include specific nodes into or exclude them from reconstructed networks with sufficient confidence and previously-acquired knowledge, there exists several design approaches for such purposes within the proposed framework. Within the work, we decipher the underlying design mechanisms of pairwise connectivity via dynamic threshold of linear/nonlinear relevance metrics, *i.e.* mutual information, correlation coefficient, and *P*-value; and determine regulatory orientations among genetic networks with signal processing metrics, *i.e*. phase shift and transfer gain.

With the inference procedure being transposed into a kind of MOCO problem, we might constrain the multiobjective iterative searching problems with reasonable terms from acquired knowledge, experimental conditions, and other computational considerations or decision-makers’ preferences.

We utilize the proposed method in analyzing two microarray datasets with different statistical characteristics. Thus by quantitative means, we reveal the inherent design mechanisms for genetic networks, facilitating the further theoretic analysis and experimental design with diverse biochemical aims.

For the sake of simplicity, we testify the proposed approach on a few small-scale datasets; different clustering and classification methods are beneficial and necessary as pre-process purposes on some large-scale, say more than hundreds or thousands of gene/proteins within those kinds of datasets.

## Methods

Based on probability and signal processing theories, the following section introduces a dimensionless metric for regulatory strengths and a phase-shift metric for determining regulatory orientations. For network inference, we propose a combinatorial-optimization framework for constraining the inference complexities. The framework allows the possibility of incorporating acquired knowledge and specific aims for integrative mining and analysis.

### Probability theory-based inference of biological network structures

Correlation analysis aims to reveal the strength of a linear relationship between random variables (R.V.); statistical correlation (coefficient) represents the departure of two R.V. from independence. Among the various metrics often used to measure the correlation or association, the *Pearson* product-moment correlation coefficient is applicable to some data of diverse characteristics. Normally, the correlation *ρ__X,Y__* is denoted as the covariance of two R.V. divided by the product of their standard deviations, which can be represented as [7,10,12,13]

                                 (1)

where cov indicates covariance, *E* is the expected value operator, *μ__X__* = *E*(*X*), and σ*_X_*^2^ = *E*[(*X*-*E*(*X*))^2^]=*E*(*X*^2^)-*E*^2^(*X*).

When interpreting the *Pearson* product-moment correlation coefficient, Cohen noted that the proposed interpretative criteria were arbitrary in general and that specific treatments should be adopted for specific cases in those ranging from physics to other social sciences [22]. Apart from the parametric statistic, nonparametric correlation metrics such as the *χ*^2^ test, Spearman’s *ρ*, and Kendall’s *τ* are proposed, and those metrics can be applied to problems of diverse nonnormal distributions [23].

### Information-theoretic inference of biological network structures

To quantify the mutual dependence of two R.V., mutual information is frequently adopted as an alternative in information-theoretic applications, in addition to the above metric. The mutual information of two discrete R.V. can be defined as [24],

                             (2)

where *p*(*x*, *y*) denotes the joint probability distribution of *X* and *Y*, and *p*__1__(*x*) and *p*__2__(*y*) represents the marginal probability distributions of *X* and *Y* respectively. The measure normally adopts the well-defined form *I*(*X*, *Y*,* b*), where *b* denotes the base. In general, a base of 2 can be specified since that is the common unit of the bit. Thus, for analysis within this context, we consistently use the base of 2.

### Associativity measure for describing regulatory connectivity

The above-described measures illustrate the correlation and dependence relationships of R.V. Normally, these R.V. characterize different entities within a system. The interconnections in the biological network can be weighted by the probability of association between the pairs being investigated [25]. Since the above metrics, *i.e.* the *Pearson* product-moment correlation and mutual information are dimensionless vector quantities; we introduce an associativity measure (AM) for illuminating the connectivity between candidate pairs. Within this uniform measure, the quantities of mutual information and correlation metrics can be projected onto the orthogonal coordinates of a 2D plane. The metric is represented in a formal term as,

                 (3)

where *MI__i__* and *Cor__i__* denote the mutual information and correlation quantities respectively; *ω*_*_i_*_1__ and *ω*_*_i_*_2__ represent the weights of both quantities; *α__i__* is the phase difference for the *i *th pair candidate; and *N* is a set of natural numbers. Note that the weights here aim to leverage any possible asymmetric distribution within the datasets of the above subterms *MI__i__* and *Cor__i__*. The weights can be derived from previously-acquired knowledge or from a specific theoretical hypothesis, *e.g.* the respective centroids of datasets.

### Phase-shift metric for determining regulatory directions

**Figure 9:**
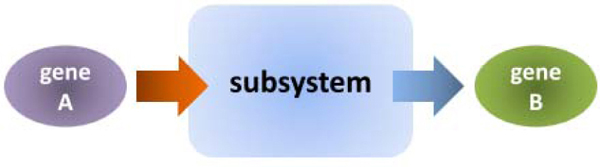
Each pairwise association might be modularized as a subsystem with the expression patterns serving as input and output signals.

Currently, most gene expression profiles are discrete time-series data. The data samples are diverse expression densities measured at multiple time points, and the data intervals represent the sampling periods. When *n* samples are compared, a total of *n*(*n*-1)/2 pairwise comparisons are obtained. Butte *et al.* utilized a type of signal processing method to cluster and compare the similarity of expression profiles [26]. For every potential pairwise regulation, the activities of the investigated genes can be modularized as a subsystem. Their expression patterns might be viewed as input and output signals, as shown in Figure 9.

For each pair, the coherence, gain, and phase shift might be calculated by discrete Fourier transform (DFT) of the inputs and outputs. The coherence of signals *a* and *b* is a function of the power spectral density (PSD) and the cross power spectral density (CPSD), defined as below,

                                               (4)

where *PSD__aa__*(*f*), *PSD__bb__*(*f*), and *CPSD__ab__*(*f*) measure the PSD and CPSD of the associated pairwise signals. The symbol *f* represents a frequency-domain metric. Normally, signals *a* and *b* are of the same length. A coherence of 1 represents a scalar multiples relationship between two investigated signals, while 0 indicates that such a relationship is not linearly related. The transfer function (TF) between two associated input/output signals measures the signal amplification and related time lag/latency properties, which are defined as,

                                                        (5)

The regular transfer functions will be of the complex-valued form, the arctangents of which are the corresponding transfer phases (TP). The absolute values denote the related transfer gains (TG), and both metrics are represented as,

                                           (6)

                                              (7)

Theoretically, the TP illustrates the phase shift between the investigated pairwise signals, *i.e.* the input and output. The phase shift ranges might be allocated within -π to π, where -π represents a phase lead of half a wavelength and π denotes a phase lag of half a wavelength. Whether the input signals are amplified or not is not illuminated at the output by the transfer gain and determines the related degrees at different frequencies. The larger the ratio, the less energy is lost by the output. Note that at different frequencies, the transfer phase and relative transfer gain might differ from each other. An effective evaluation criterion for these metrics is the related coherence, namely, at frequencies where the coherence values are high, the corresponding transfer phases and gains are much more reliable than others.

The advantages of such metrics lie in the flexible and quantitative characteristics of determining the regulatory delay via dynamic threshold. Factual regulatory mechanisms have multiple possibilities, and inherent regulatory delay effects might vary during the whole biological processes. The phase-shift metric determines such possibilities underlying regulatory mechanisms in a quantitative manner. The advantages include the inherent capabilities of integrating *a priori* biological knowledge. This kind of knowledge-based inference method avoids redundant false-positive connectivities within pairwise candidates.

Such dynamic threshold is applicable to the majority of problems facing theoretical and experimental biologists. Since regulatory connectivity underlying pairwise candidates may differ in diverse processes or at different sampling times, systematic and quantitative determination of these regulations with empirical and theoretical knowledge will be much more effective than those generated by most currently-available computational approaches [17]. Such types of flexible network connectivities and regulations characterize major regulatory processes from the perspectives of information and signal processing theories.

### A MOCO pattern for constraining computational complexities

In the following sections, we extract inherent regulations and decipher network structures by introducing a pairwise gene hierarchy criterion (PGHC) for classifying possible gene pairs into three major groups as follows.

(1) Authentic Pairwise Genes (APGs): These include pairs with mutual information values and correlation coefficients larger than specific thresholds. Moreover, the corresponding *P* value resides in the confidence interval, namely, smaller than 0.05.

(2) Questionable Pairwise Genes (QPGs): These include pairs that do not satisfy both of the thresholds mentioned above. The group contains pairs of two classes. One class has pairs with mutual information larger than specific thresholds but satisfies neither the criteria of correlation coefficients nor *P* values. The other class includes pairs with correlation coefficients larger than specific thresholds and with *P* values residing in the confidence interval but the related mutual information does not satisfy specific thresholds.

(3) Unauthentic Pairwise Genes (UPGs): These include those pair candidates that do not satisfy any criteria of the APGs or QPGs defined above.

The QPGs actually act as a subsidiary candidate pool for the APGs in case the empirical thresholds are set too high to extract structures merely from the APGs. Under such conditions, the QPGs will be ranked according to mutual information values, correlation coefficients, and *P* values. Optimal pairs will be allocated to the APGs to refine the former network connectivity. The algorithm for the supervised PGHC is shown in table 1.

**Table 1:** Algorithm: Pairwise Gene Hierarchy Criterion

**Input:**
all pairwise gene candidates GPs; initial MI threshold MIth = MI's centroid; initial CC threshold CCth = CC's centroid; increments *δ*_mi_, *δ*_cc_ for MI and CC.
**Output:**
classified APGs, UPGs and QPGs.
**while** count(GPs)>0 **do**
1. construct APGs, QPGs using initial MIth, CCth and *P*-value; 2. group the others into UPGs; **if** (APGs' undersized) && count(QPGs)>0 **then do**
MIth=MIth-*δ*_mi_ & CCth=CCth-*δ*_cc_; continue Step 1 for QPGs & obtain Δ_APGs_ and Δ_UPGs_; APG=APGs+Δ_APGs_ & UPGs=UPGs-Δ_UPGs_.
**elseif**(APGs' oversized) **then do**
MIth=MIth-*δ*_mi_ & CCth=CCth+*δ*_cc_; continue Step 1 for APGs & obtain Δ_APGs_ and Δ_UPGs_; APG=APGs-Δ_APGs_ & UPGs=UPGs+Δ_APGs_.
**endif**
**end**

Thus, network reconstruction might be transformed into a class of MOCO problems [10,12,13]. The optimization objectives include first reaching suitable thresholds for mutual information and correlation coefficient to maximize the feasible components in the APGs. The inference might be carried out with much more confidence and reliability. The second objective is to maximize the UPGs. The larger the UPGs, the fewer the problems faced during further solution searching. This decreases the feasible solution space for subsequent computations. In addition, the following relative constraints exist. There are nonnegative constraints for the sizes of groups, and the total number of pair candidates is fixed, *i.e.* the valid combinatorial space is limited. The gain thresholds for guaranteeing valid network connectivity and previously-acquired biochemical knowledge and different experimental conditions constitute other prominent constraints for the reconstruction process. The MOCO paradigm is described as follows,

              (8)

where *F__i__* is the multiobjective function set; *S*__1__ is the set of feasible group combinations for APGs, QPGs, and UPGs; *S*__2__ is the number set of all gene pairs (*S_2_* = {*n*(*n*-1) / 2}, *n* is the total number of genes); *S*__3__ is the set of necessary gain constraints (GC); and *S*__4__ is the set of possible constraints from acquired biological knowledge (ABK).

Recently quite a few authors have argued the necessity of incorporating the preferences of decision-makers (DM) into MOCO solution selection [27-29]. For the problem under investigation, the DM’s preferences mainly stem from the GC (*S*__3__) and ABK (*S*__4__) illustrated above.

In cases governed by lower thresholds of mutual information and correlation metrics, APGs will form the group with the maximum components within the total pair candidates. On the other hand, with the heightened thresholds, many more pairs might be grouped into UPGs. This reduces the computational complexity for network reconstruction since APGs have fewer components in such situations. If APGs are classified with above-normal sizes, the reconstructed network will be densely connected and will have much more redundancies. On the contrary, a sparsely connected structure will be inferred with an undersized candidate group of APGs.

Since biological theoreticians and experimentalists may vary specific mutual information and correlation thresholds to incorporate empirical or concrete knowledge into the reconstruction procedures, the underlying coordination approaches via the MOCO framework might be feasible and significant, especially for those containing pivotal structural connectivity or for specific analysis purposes.

**Figure 10:**
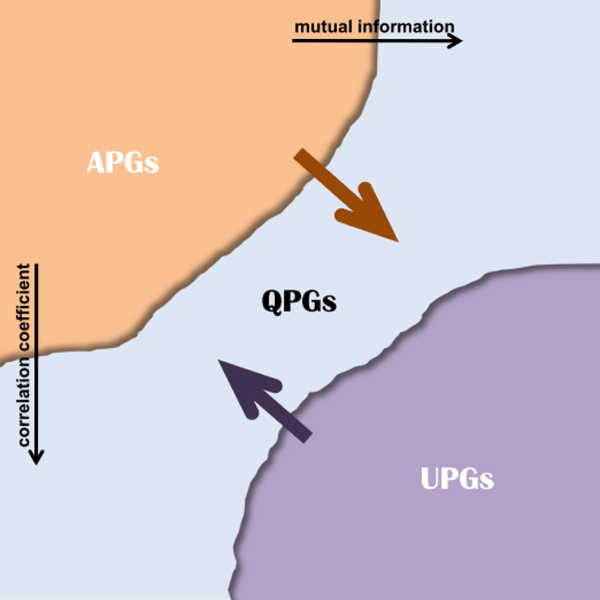
**Schematic representation of the MOCO problem by dynamic thresholding of mutual information and correlation metrics.** Total pairs are classified into APGs, QPGs and UPGs. The upper rightward horizontal arrow represents dynamic thresholding by mutual information, and the left descending arrow is for thresholding of the correlation measure.

The APGs, QPGs, and UPGs engender the underlying evolutionary mechanisms with respect to dynamic threshold by the above metrics and related biochemical knowledge, as shown in Figure 10.

## Competing interests

The authors declare that they have no competing interests.

## Authors' contributions

BHT proposed the methods, performed the analysis and composed the work; XCW and GT gave advice and proof-checked the work; SSC commented on the methods and the writing; QJ and BRS led the project and coordinated the research progress.

## Supplementary Material

Additional file 1 The calculated mutual information matrix for 276 gene pairs from the 24 cell-cycle genes. Click here for file

Additional file 2 The descending-order sorted mutual information, correlation coefficient and corresponding P-value statistics. Click here for file

Additional file 3 The three-dimensional distribution for authentic (APGs), questionable (QPGs), and unauthentic pairwise genes (UPGs). Click here for file

Additional file 4 The phase-shift statistics for the group APGs. Click here for file

Additional file 5 Associativity measure statistics for the group APGs from the Saccharomyces cerevisiae cell cycle microarray dataset. Click here for file

Additional file 6 Mutual information matrix for the triplicate MOTL4 microarray experiments. Click here for file

Additional file 7 The descending-sorted mutual information, correlation coefficient and corresponding P-value statistics. Click here for file

Additional file 8 The three-dimensional distribution for authentic (APGs), questionable (QPGs), and unauthentic pairwise genes (UPGs). Click here for file

Additional file 9 The phase-shift statistics for the group APGs. Click here for file

Additional file 10 The constructed genetic map with gain threshold at 1. Click here for file

Additional file 11 Associativity measure statistics for the group APGs in the human cancer MOTL4 cell cycle microarray dataset. Click here for file
