# Supplementary material for: Computational inference and analysis of genetic regulatory networks via a supervised combinatorial-optimization pattern
Source: BMC Syst Biol. 2010 Sep 13;4(Suppl 2):S3. doi: 10.1186/1752-0509-4-S2-S3 (PMC2982690; doi:10.1186/1752-0509-4-S2-S3)
Supplement: Additional file 1 — The calculated mutual information matrix for 276 gene pairs from the 24 cell-cycle genes. [file 1752-0509-4-S2-S3-S1.doc]

**
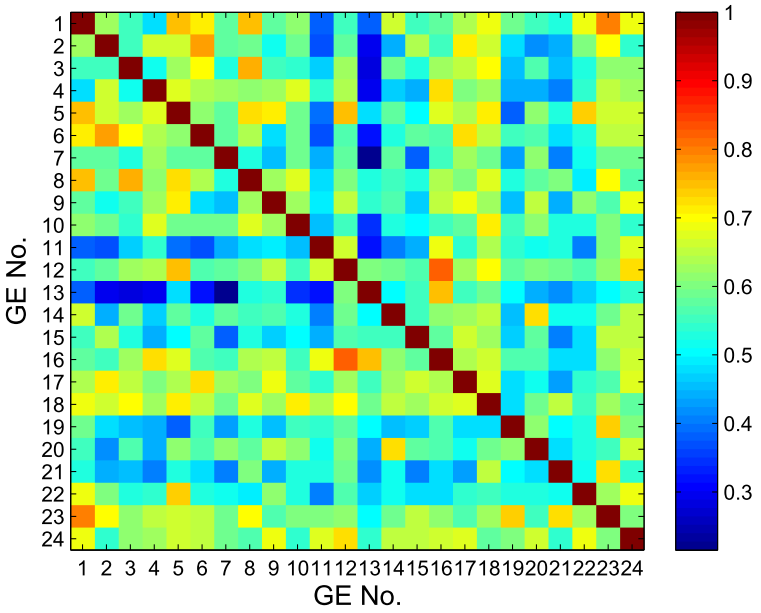
**

**Additional Figure 1-A.** The calculated mutual information matrix for 276 gene pairs from the 24 cell-cycle genes. The diagonal elements are all equal to one since mutual information is maximized for measuring the totally same variables. The mutual information matrix is nonnegative (*I*(*X*;*Y*)≥0) and symmetric (*I*(*X*;*Y*)=*I*(*Y*;*X*)).
