# Supplementary material for: Computational inference and analysis of genetic regulatory networks via a supervised combinatorial-optimization pattern
Source: BMC Syst Biol. 2010 Sep 13;4(Suppl 2):S3. doi: 10.1186/1752-0509-4-S2-S3 (PMC2982690; doi:10.1186/1752-0509-4-S2-S3)
Supplement: Additional file 2 — The descending-order sorted mutual information, correlation coefficient and corresponding P-value statistics. [file 1752-0509-4-S2-S3-S2.doc]

**
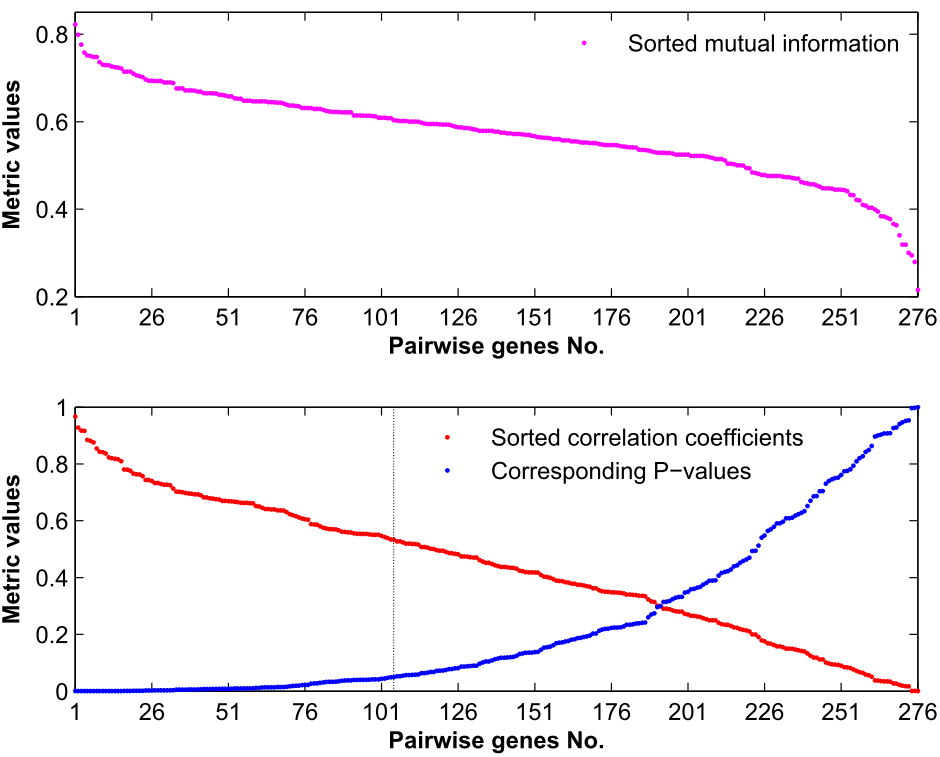
**

**Additional Figure 1-B.** The descending-order sorted mutual information, correlation coefficient and corresponding *P*-value statistics for the total pairwise candidates of the cell cycle regulatory network. As indicated by the vertical dotted line in the lower plot, there are totally 105 pairs with their *P*-values smaller than 0.05.
