# Supplementary material for: Computational inference and analysis of genetic regulatory networks via a supervised combinatorial-optimization pattern
Source: BMC Syst Biol. 2010 Sep 13;4(Suppl 2):S3. doi: 10.1186/1752-0509-4-S2-S3 (PMC2982690; doi:10.1186/1752-0509-4-S2-S3)
Supplement: Additional file 4 — The phase-shift statistics for the group APGs. [file 1752-0509-4-S2-S3-S4.doc]

**
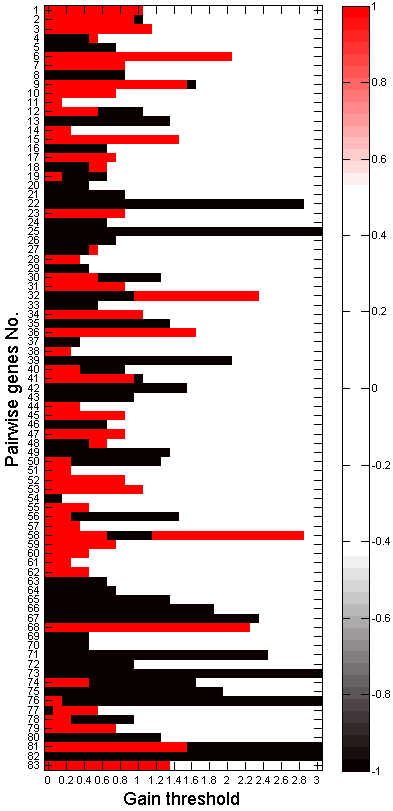
**

**Additional Figure 3-A.** The phase-shift statistics for the group APGs (totally 83 pairwise genes, sorted according to descending mutual information values of each pair), calculated based on the signal processing concepts defined above. The red part (+1) represents the leading phase shift for the related pairwise genes, the black (-1) for the lagging phase shift, and the white for those pairs without any phase shift under specific gain thresholds.
