# Supplementary material for: Computational inference and analysis of genetic regulatory networks via a supervised combinatorial-optimization pattern
Source: BMC Syst Biol. 2010 Sep 13;4(Suppl 2):S3. doi: 10.1186/1752-0509-4-S2-S3 (PMC2982690; doi:10.1186/1752-0509-4-S2-S3)
Supplement: Additional file 6 — Mutual information matrix for the triplicate MOTL4 microarray experiments. [file 1752-0509-4-S2-S3-S6.doc]

**
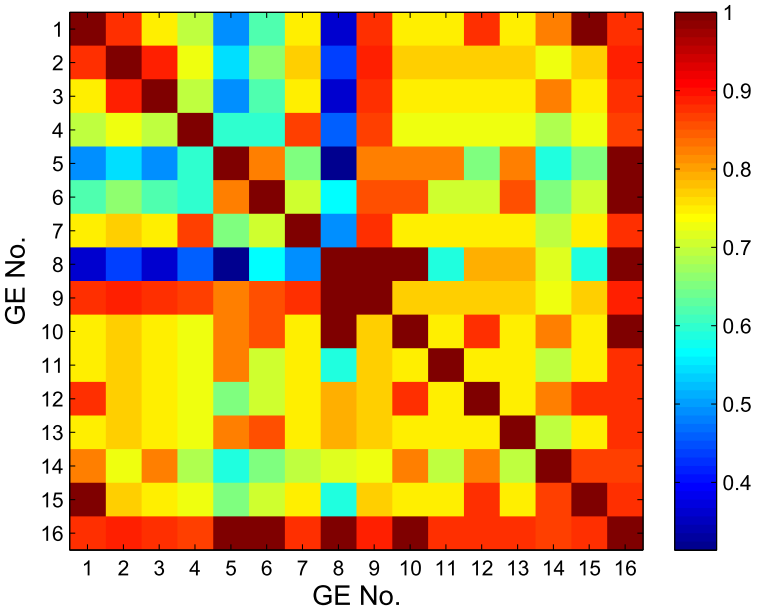
**

**Additional Figure 5-A.** Mutual information matrix for the triplicate MOTL4 microarray experiments, implemented under irradiation from 0 to 12 hours at intervals of 2 hours.
