# Supplementary material for: Computational inference and analysis of genetic regulatory networks via a supervised combinatorial-optimization pattern
Source: BMC Syst Biol. 2010 Sep 13;4(Suppl 2):S3. doi: 10.1186/1752-0509-4-S2-S3 (PMC2982690; doi:10.1186/1752-0509-4-S2-S3)
Supplement: Additional file 7 — The descending-sorted mutual information, correlation coefficient and corresponding P-value statistics. [file 1752-0509-4-S2-S3-S7.doc]

**
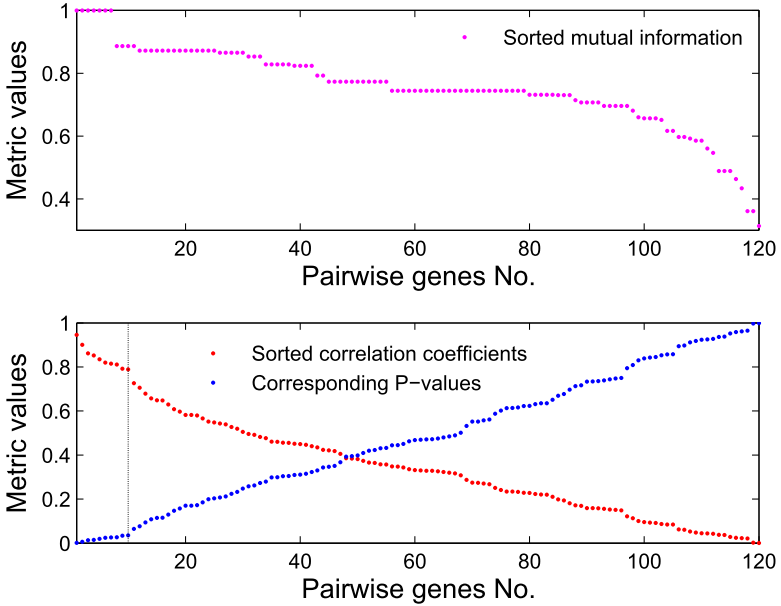
**

**Additional Figure 5-B.** The descending-sorted mutual information, correlation coefficient and corresponding *P*-value statistics for the total pairwise candidates of the multi-feedback p53 pathway. The mutual information statistics are of the homogeneous distribution among the range between 0.3134 and 1, while note that the Pearson correlation statistics only have 10 candidates with *P*-values below 0.05, indicated with the vertical dashed line in the lower subgraph.
