# Supplementary material for: Computational inference and analysis of genetic regulatory networks via a supervised combinatorial-optimization pattern
Source: BMC Syst Biol. 2010 Sep 13;4(Suppl 2):S3. doi: 10.1186/1752-0509-4-S2-S3 (PMC2982690; doi:10.1186/1752-0509-4-S2-S3)
Supplement: Additional file 8 — The three-dimensional distribution for authentic (APGs), questionable (QPGs), and unauthentic pairwise genes (UPGs). [file 1752-0509-4-S2-S3-S8.doc]

**
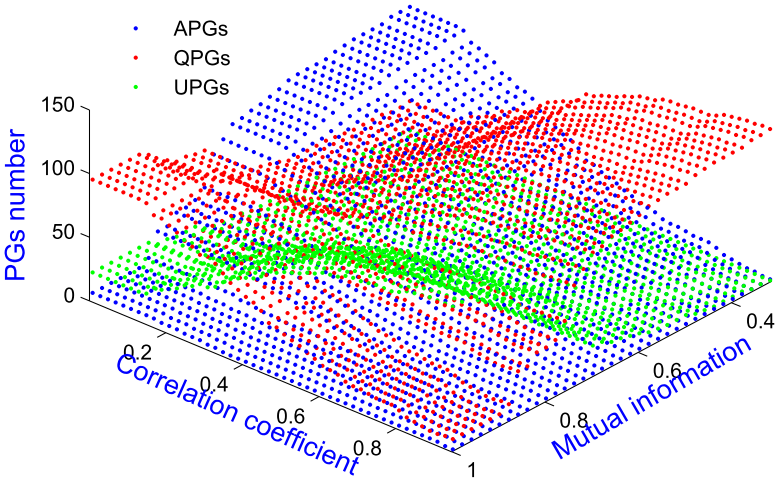
**

**Additional Figure 6-A.** The three-dimensional distribution for authentic (APGs), questionable (QPGs), and unauthentic pairwise genes (UPGs) under different thresholds of mutual information and correlation coefficient. The related *P*-value adopts 0.8 to ensure enough candidates in the APGs for the network-reconstruction. Totally, there are 120 pairs among 16 genes for the cell cycle regulatory network.
