# Supplementary material for: Computational inference and analysis of genetic regulatory networks via a supervised combinatorial-optimization pattern
Source: BMC Syst Biol. 2010 Sep 13;4(Suppl 2):S3. doi: 10.1186/1752-0509-4-S2-S3 (PMC2982690; doi:10.1186/1752-0509-4-S2-S3)
Supplement: Additional file 10 — The constructed genetic map with gain threshold at 1. [file 1752-0509-4-S2-S3-S10.doc]

**
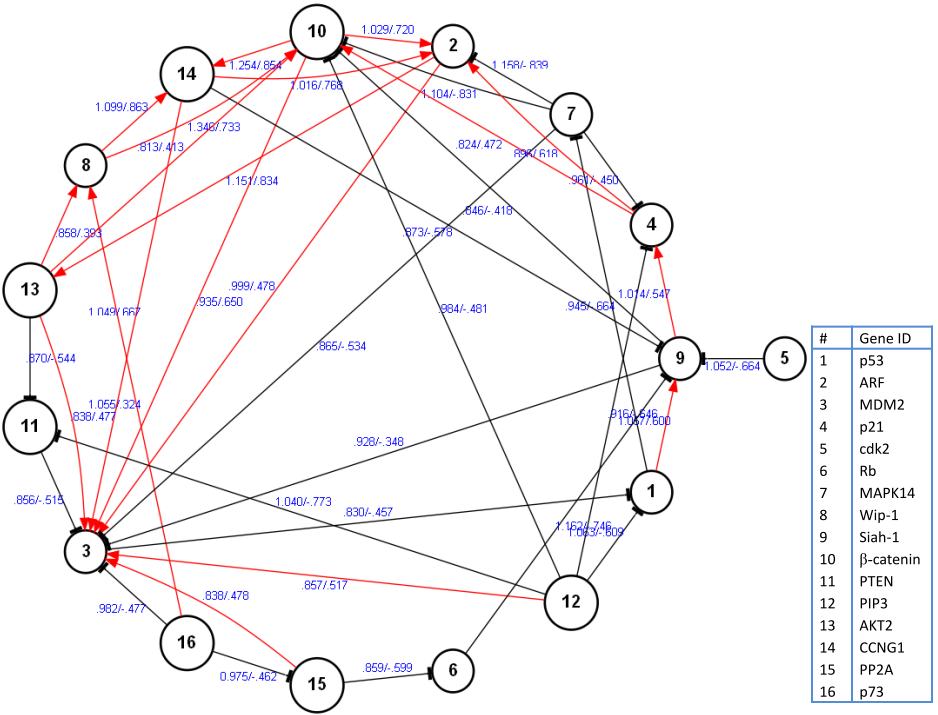
**

**Additional Figure 8-A.** The constructed genetic map with gain threshold at 1. As depicted in the graph, #5 (cdk2) and #6 (Rb) are the weak-connected nodes, #3 (MDM2) and #10 (β-catenin), *etc*. are the strong-connected ones under the current gain threshold.
