# Supplementary material for: Computational inference and analysis of genetic regulatory networks via a supervised combinatorial-optimization pattern
Source: BMC Syst Biol. 2010 Sep 13;4(Suppl 2):S3. doi: 10.1186/1752-0509-4-S2-S3 (PMC2982690; doi:10.1186/1752-0509-4-S2-S3)
Supplement: Additional file 11 — Associativity measure statistics for the group APGs in the human cancer MOTL4 cell cycle microarray dataset. [file 1752-0509-4-S2-S3-S11.doc]

**
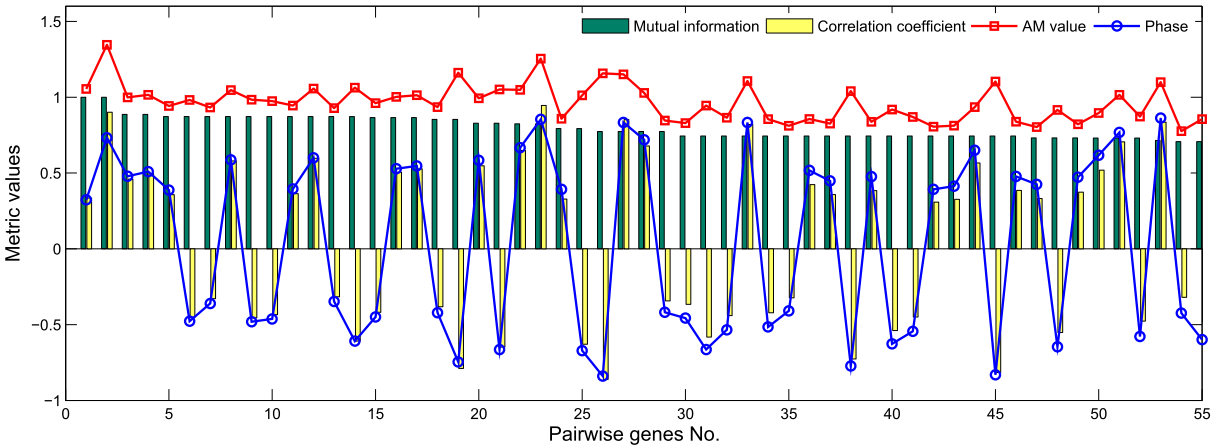
**

**Additional Figure 8-B.** Associativity measure statistics for the group APGs in the human cancer MOTL4 cell cycle microarray dataset. The statistics are calculated based on the concepts defined in the methodology section.
